# Fabrication and Cytocompatibility of *In Situ* Crosslinked Carbon Nanomaterial Films

**DOI:** 10.1038/srep10261

**Published:** 2015-05-28

**Authors:** Sunny C. Patel, Gaurav Lalwani, Kartikey Grover, Yi-Xian Qin, Balaji Sitharaman

**Affiliations:** 1 Department of Biomedical Engineering, Stony Brook University, Stony Brook, NY 11794-5281, USA

## Abstract

Assembly of carbon nanomaterials into two-dimensional (2D) coatings and films that harness their unique physiochemical properties may lead to high impact energy capture/storage, sensors, and biomedical applications. For potential biomedical applications, the suitability of current techniques such as chemical vapor deposition, spray and dip coating, and vacuum filtration, employed to fabricate macroscopic 2D all carbon coatings or films still requires thorough examination. Each of these methods presents challenges with regards to scalability, suitability for a large variety of substrates, mechanical stability of coatings or films, or biocompatibility. Herein we report a coating process that allow for rapid, *in situ* chemical crosslinking of multi-walled carbon nanotubes (MWCNTs) into macroscopic all carbon coatings. The resultant coatings were found to be continuous, electrically conductive, significantly more robust, and cytocompatible to human adipose derived stem cells. The results lay groundwork for 3D layer-on-layer nanomaterial assemblies (including various forms of graphene) and also opens avenues to further explore the potential of MWCNT films as a novel class of nano-fibrous mats for tissue engineering and regenerative medicine.

Carbon nanomaterials such as fullerenes, carbon nanotubes (CNTs) and graphene possess unique physiochemical properties^1^,^2^, and thus, assembly of these nanoscale building blocks into two dimensional (2D) macroscopic coatings and films that harness these properties may lead to high impact biomedical applications. Over the last decade, carbon nanomaterials have been identified as a platform technology for tissue engineering by providing matrix reinforcement to polymeric scaffolds and as substrates for electrically stimulated osteo-conduction and for neuronal network formation^3^. However, compared to 2D macroscopic films and coatings of carbon nanomaterials for electronics and energy storage applications^4^, very few studies (all *in vitro*) have investigated the potential and suitability of carbon nanomaterial thin films for biomedical applications^5^,^6^,^7^. Chemical vapor deposition (CVD), spray (electrospray and air-pressure driven) and dip coatings, and vacuum filtration are few of the methods that have been employed for the fabrication of 2D carbon nanomaterial films^8^. While the suitability of these methods for material science or electronic application has been examined, the adaptation of these techniques for biomedical applications still needs thorough evaluation.

Two issues need to be assessed for biomedical applications of 2D carbon nanomaterial films: 1) the suitability of the fabrication method, and 2) biocompatibility of carbon nanomaterial thin films fabricated using each method. CNT forests or single layer graphene coatings fabricated using CVD have been investigated as cell substrates^9^; and have been investigated for their ability to differentiate stem cells to bone lineages^10^,^11^. However, CVD method requires very specific substrates for nanomaterial film growth or deposition. For instance, direct growth of carbon nanotube forests^12^ or graphene^13^ coatings by CVD requires substrates that can withstand high temperatures and pressures. Although, micron-scale thick films of CNTs^14^ or graphene can be fabricated by vacuum filtration^11^, this method requires flat substrates to maintain their structural features, and cannot be easily applied to irregular or round shapes such as a hip implant ball head. Spray coating techniques (e.g. airbrushing, electro-spraying, plasma spraying) do allow optimal carbon nanomaterial coatings on irregular, non-flat substrates. A limitation of these spray coating techniques, and indeed all the above techniques is the absence of strong chemical bonds between the individual nanomaterials. Therefore, the structural integrity of films and coatings relies mainly on physical entanglement of the nanoparticles, or weak Van der Wall forces. Thus, these films and coatings could be prone to disassociation under compressive flexural or shear forces that biomaterials devices and implants experience under physiological conditions.

Few studies have also investigated the *in vitro* cytocompatibility of carbon nanomaterials (graphene and carbon nanotubes) thin films for tissue engineering applications fabricated by some of the above methods. Carbon nanotube and graphene substrates, prepared by CVD^5^ and spray coating^15^, have been reported to enhance osteogenesis, and upregulate bone matrix mineralization in human mesenchymal stem cell populations. Vacuum filtration-based graphene^11^ and carbon nanotubes^16^ films have shown cytocompatibility towards mouse fibroblasts and enhanced matrix production by osteoblastic cells, respectively. However, the most densely packed films of carbon nanomaterials, fabricated by vacuum filtration, have been reported to elicit cytotoxic response; attributed to loose nanomaterials that peel away from the films and get uptaken by osteoblasts^16^.

We have recently developed a facile low-cost chemical synthesis protocol that allows the assembly of sp^2^ hybridized carbon nanostructures such as fullerenes, carbon nanotubes and graphene into free-standing, chemically-crosslinked macroscopic all-carbon architectures^17^. The protocol involves radical-initiated thermal crosslinking and annealing of sp^2^ hybridized carbon nanostructures. The objective of this study was to adapt an air-pressure driven spray coating technique to develop an innovative *in situ* method to fabricate more robust, chemically-crosslinked all carbon multi-walled carbon nanotube (MWCNT) films. As novel nanofiber mats, we have also evaluated *in vitro* the cytocompatibility of crosslinked MWCNTs films towards their development as scaffolds for tissue engineering applications, and coating for biomedical implants.

## Results and Discussion

### Physicochemical Characterization of Crosslinked MWCNT Coatings

**Figure 1:**
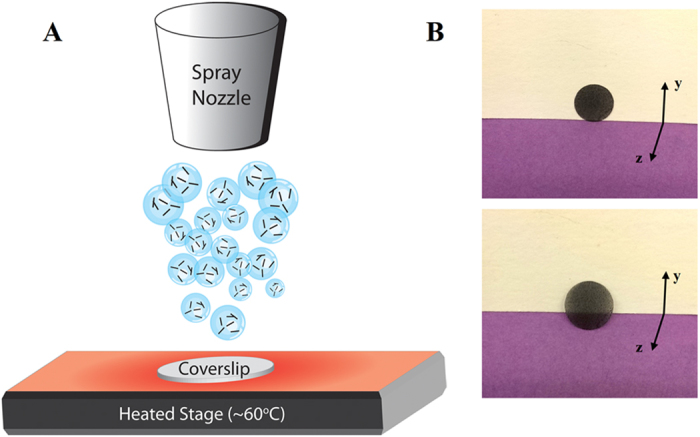
Fabrication of crosslinked carbon nanomaterial films. (**A**) Illustration of *in situ* crosslinking process. (**B**) Photograph of film standing vertically (top) and tilted to show transparency (bottom).

Figure 1A depicts the fabrication process. An air pressure driven device sprayed the nanomaterial and benzoyl peroxide solution onto a coverslip heated to 60 °C (Fig. 1A). The MWCNTs completely coated the coverslips (Fig. 1B, top) and were semi-transparent (Fig. 1B, bottom). The spraying method leads to the generation of heterogeneously-sized droplets of MWCNT and benzoyl peroxide which deposit onto the heated coverslip. The solvent (ethyl acetate) immediately evaporates, and simultaneously the free radical crosslinking process is initiated which in turn leads to the *in situ* crosslinking of MWCNTs, and fabrication of the films. For all characterization and cell studies, a preset volume and mass of nanomaterial solution was utilized for the fabrication of each film.

**Figure 2:**
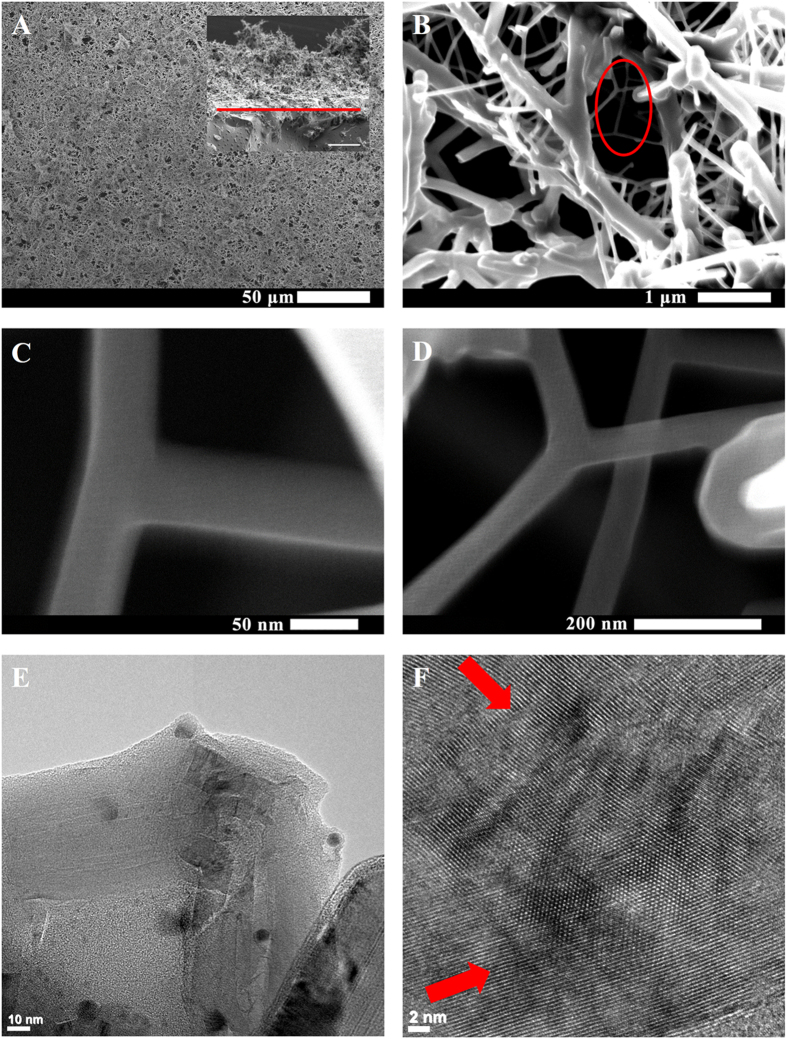
Representative SEM and TEM micrographs for 1:4 MWCNT films. (**A**) Overview micrograph of crosslinked MWCNT coating with a cross-sectional inset in the upper right (scale bar 100 μm). Red line denotes the surface-nanomaterial interface. (**B**) Junctions between nanotubes, shown in red oval (**B**) and magnified in (**C**) and (**D**), suggest crosslinking. (**E**) TEM image of a single crosslink junction with (**F**) the directions of intersecting MWCNT lattice shown by red arrows.

Low magnification SEM analysis showed that all films created a continuous coating on 12 mm diameter glass coverslips with a micro porous network (Fig. 2A). The films had high surface roughness with a mean height of 75 μm (Fig. 2A inset). Ultra-high resolution SEM showed MWCNT networks with connectivity, micro- and nano-porosity with numerous junctions (Fig. 2B) of individual MWCNT and bundles crosslinking with each other (Fig. 2C-D). TEM analysis suggests junctions between two MWCNTs (Fig. 2E) leading to a checkerboard pattern (Fig. 2F) similar to previously reported chemically crosslinked MWCNTs^17^. Atomic force microscopy revealed a high root mean square (rms) area surface roughness of 730 nm (S.D. 124 nm, n = 3).

**Figure 3:**
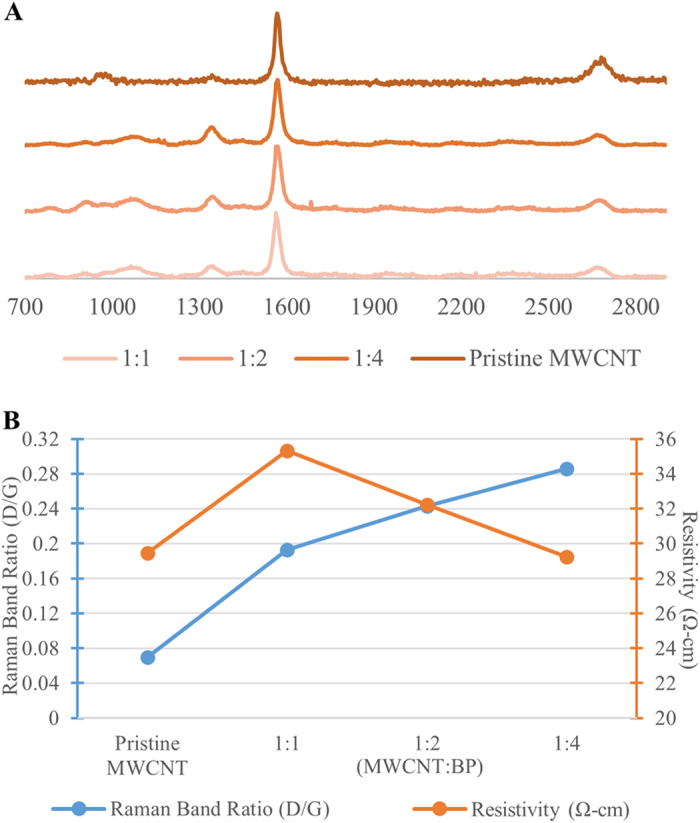
**A**) Representative Raman spectroscopy for MWCNT crosslinked films with three different mass ratios of MWCNT:BP (1:1, 1:2, 1:4). **B**) MWCNT crosslinked films sheet resistivity and tubular defects analyzed by a four point resistivity system and Raman spectroscopy (D/G bands) respectively.

Normalized Raman spectra of crosslinked MWCNT films are presented in Fig. 3A. Each mass ratio showed the characteristic Raman peaks of MWCNT with D, G, and G’ bands at ~1345 cm^−1^, 1560 cm^−1^, and 2670 cm^−1^, respectively^18^. Pristine graphitic network is characterized by the G band (intensity represented by I_G_) generated by in-plane vibrations of C=C carbon atoms and the D band (intensity represented by I_D_) is generated by structural defects or disorder features in graphitic network^18^. The I_D_/I_G_ ratio increased with increase in MWCNT: BP ratio (Fig. 3B). Further, weakly defined peaks at 802 cm^−1^ to 915 cm^−1^ were observed and assigned to C-O-C bond vibrations and asymmetrical stretching, respectively^19^. These peaks imply presence of covalent carbonyl functional groups most probably formed during the crosslinking reaction.

Electrical resistivity measurements allow evaluation of the changes in the electrical properties of the MWCNT after the crosslinking reaction. The changes provide surrogate information about the interconnectivity between the MWCNTs^20^. Figure 3B shows the bulk electrical resistivity of the MWCNT films as a function of MWCNT: BP ratio. We found pristine MWCNT coatings to have a resistivity of 29.45 Ω-cm. Adding BP (MWCNT:BP) to samples lead to an initial increased in sheet resistivity to 35.3 Ω-cm for 1:1 (MWCNT:BP) mass ratios and reduction thereafter in sheet resistivity to 29.2 Ω-cm for 1:4 (MWCNT:BP) mass ratios.

Nanoindentation was performed to characterize the mechanical properties of spray-coated chemically-crosslinked MWCNT (1:4) films. Spray coated pristine MWCNT films (without chemical crosslinking) were used as controls. The nanoindentation protocol yielded the values of elastic modulus (Er) and hardness (H) of the films, and are summarized in Table 1. Representative force-displacement curves for the chemically-crosslinked MWCNT and pristine MWCNT films are presented in Supplemental Figure 1. The median Er value of the chemically-crosslinked MWCNT films (mdn = 376 MPa, μ = 424 MPa, S.D. = 287 MPa) were ~232% greater than that of pristine MWCNT (mdn = 162 MPa, μ = 232 MPa, S.D. = 299 MPa) (p < 0.0001). The crosslinked MWCNT films also exhibited statistically significant (p < 0.0001) ~242% increase in hardness (mdn = 5.15 MPa, μ = 5.83 MPa, S.D. = 3.84 MPa) compared to the pristine MWCNT films (mdn = 2.13 MPa, μ = 2.37 MPa, S.D. = 1.61 MPa).

While the focus of the current study has been on fabrication of all carbon films using MWCNTs as starting material, an advantage to this method lies in its versatility. It can easily be adapted for different sp^2^ hybridized allotropes of carbon including, but not limited to, various types of graphene (e.g graphene nano-onions, nanoribbons and nanoplatelets. See Supplemental Figure. 2). This method has four additional advantages: (1) The method should be suitable for a wide variety of substrates (e.g. flexible, irregular or round shaped). The substrates need to be thermally stable up to 60 °C and compatible to organic solvents. Comparatively, CVD based films can be grown on a wide variety of substrates, and vacuum filtration films can be fabricated at low temperatures on flat substrates with organic solvents; however, to the best of our knowledge, neither technique allows the flexibility of substrates, solvents and low temperature. (2) This method allows facile control of film thickness. CVD allows deposition of monolayer film of vertically-aligned carbon nanotube forests, or few-layer film of randomly-aligned carbon nanotubes^21^ or single and multi-layered graphene^13^. Its capabilities to create thick films still need to be thoroughly explored. Vacuum filtration typically allows maximum film thicknesses of 10–150 μm for MWCNT and graphene, since the passage of the filtrate through the filter membrane restricts flow as the film gets deposited on the substrate^11^,^22^,^23^. Spray coating of carbon nanotubes onto substrates show sparse network formation^24^ which can be a hindrance to create films of controllable thickness. Our results indicate that covalent bonding between the MWCNT when combined with spray coating allows for a layer-by-layer assembly of thick coatings (>100 μm in thickness). (3) This method yields coatings with nano- and micro-pores and high surface roughness; advantageous for applications such as biosensing which require high charge storage capacity^25^,^26^ and self-cleaning hydrophobic substrates^27^. Methods such as vacuum filtration and CVD are more suitable for applications that require homogenous roughness coating^28^. Even though spray coating allows coating of irregular shapes, and ability to create a continuous network (Fig. 2A), the high surface roughness (~730 nm) using current air spray method warrants exploration of other spray coating techniques. The droplet size inhomogeneity (as depicted in Fig. 1A), and nanomaterial aggregation may be responsible for the increased roughness^29^,^30^. For applications requiring crosslinked MWCNT films with a smoother surface, more homogenous spray coating techniques such as ultrasonic spray coating could be employed. Ultrasonic spray coating has recently shown the ability to create functionalized-SWCNT films of 3 nm average surface roughness. In comparison, vacuum filtration of carbon nanotubes on mixed cellulose ester and transferred to a smooth silicon wafer resulted in 8 nm r.m.s surface roughness^28^. (4) The chemical crosslinking of MWCNTs, substantially enhances the mechanical properties of the films and thus, their structural stability compared to pristine non-crosslinked MWCNT films. This enhancement should prevent the films from disintegration under compressive flexural or shear forces under physiological conditions. Indeed, the crosslinked MWCNT films remained intact during the entire duration (5 days) of static culture experiments. The stability of the films under dynamic conditions still needs to be evaluated. The mean elastic modulus values for crosslinked MWCNT films were comparable and in the same orders of magnitude as polyelectrolyte layer-by-layer SWCNT films^31^, and MWCNTs crosslinked through chemical reaction of functional groups on their surface^32^. However, the elastic modulus of the crosslinked MWCNT is an order of magnitude lower than bucky-paper formed by high pressure compression of CVD synthesized MWCNT films^33^. Though here, the porous structure and control over the porosity of the films also provides a tailorable framework for incorporation of polymers and ceramics to develop novel mechanically-reinforced composites.

Carbon nanotubes are known to be excellent conductors of electricity, and disruptions (due to functionalization of structural defects) to the sp^2^ carbon network are known to decrease electrical conductivity of carbon nanotubes^34^. Although 1:1 (MWCNT:BP) samples showed greater sheet resistivity than pristine MWCNT coatings, we interestingly observed a decrease in sheet resistivity with increase in the defect sites in the MWCNT films to the point of recovery by 1:4 (MWCNT:BP) (Fig. 3B). This trend can be attributed to the increased interconnectivity between MWCNT bundles in a less densely packed system^35^. The crosslinked MWCNT films exhibit resistivity similar to previously reported spray coated pristine MWCNT with resistivity values sufficient for applications in electrostatic dissipation and transfer^36^. Although the results suggest that conductivity can be increased by increasing BP concentration for spray coating, high concentrations of BP may cause excessive oxidation of the nanotubes and possible decrease in conductivity. We observe an increasing amount of sidewall defect formation in the crosslinked carbon nanotube samples and increased electrical resistivity, when compared to the pristine MWCNTs, for 1:1 and 1:2 samples, suggesting that MWCNT interconnectivity and sidewall defects contribute to the electrical properties of these materials. Furthermore, termination reactions involving the benzoyloxyl radicals at MWCNT radical sites may increase as a function of BP concentration as this has been observed in oxidation of olefins by high concentrations of BP^37^. The decrease in conductivity due to oxidation of the nanotubes could be mitigated by treating the MWCNT substrates with reducing agents such as hydrazine hydrate^38^ or more biocompatible solutions such as ascorbic acid^39^.

### Cytocompatibility and Cytotoxicity

The potential *in vivo* biomedical applications of the MWCNT mats require thorough evaluation of their biocompatibility. *In vitro* cytotoxicity studies are typically the first step before more elaborate and costly *in vivo* animal biocompatibility experiments. Since the MWCNTs mats could be utilized as coatings for orthopedic implants and devices or bone tissue engineering scaffolds, *in vitro* interactions with human adipose derived stem cells (ADSCs) is investigated^40^. Adipose tissue is a good source for multi-potent mesenchymal stem cells; giving greater cell yields with less invasive extraction procedures^41^ and good immunosuppressive properties for mitigating graft-host disease^42^. Proliferation and cytotoxicity assays were performed on ADSCs incubated on crosslinked MWCNT films (1:4 of MWCNT:BP) and glass coverslips (control). MTS assay is a measures the conversion of tetrazolium salt to water-soluble formazan crystals by mitochondrial processes in proliferating cells^43^. The release of lactate dehydrogenase (LDH) from compromised cell membranes of dying cells was measured by the LDH toxicity assay. LDH causes oxidation of lactate to form pyruvate which coverts tetrazolium salt to formazan crystals for colorimetric assessment of LDH release by absorbance spectroscopy^44^. MTS and LDH assays were performed at day 1, 3, and 5 time points. Cytotoxicity of crosslinked MWCNT substrates are normalized to the LDH release of the positive control (100% dead cells by lysis buffer). Live cells on glass coverslips were used as the baseline control for basal LDH release.

**Figure 4:**
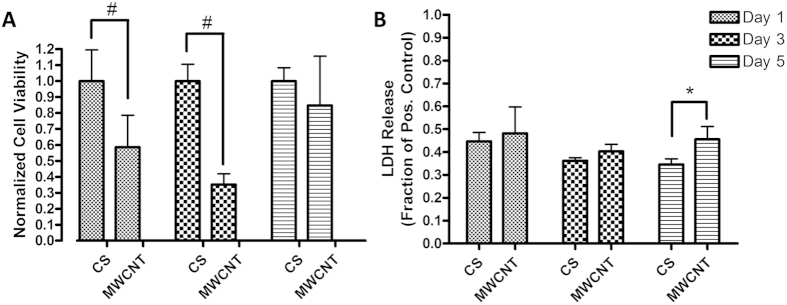
(**A**) Cell proliferation assessed by MTS assay and (**B**) cytotoxicity assessed by LDH release for ADSCs 1:4 MWCNT:BP crosslinked substrates (MWCNT) and glass coverslips (CS). Data are presented as mean ± SD (n = 6 per group, ^#^indicates p < 0.001 and *indicates p < 0.01)

Figure 4A shows cell proliferation normalized to the control glass coverslips for ADSCs. The initial cell attachment (day 1) on crosslinked MWCNT substrates was approximately 59% of the control group (p < 0.001) (Fig. 4A). At the day 3 time point, there were still significantly less cells on the crosslinked MWNCT substrates (36%, p < 0.001) relative to the control group (Fig. 4A). The cells also proliferated slower from day 1 to day 3 on the crosslinked MWCNT substrates with a 23% decrease in proliferation, compared to the control. Interestingly, the difference in cell viability at the day 5 timepoint was not significant and recovered to 85% of the value as compared to the control coverslips.

Figure 4B shows the cell death on crosslinked MWCNT substrates. The results are normalized to a positive control of 100% dead cells by using an LDH assay lysis buffer. The ADSCs grown on coverslips released approximately 35% and 45% of LDH at days 1 and 3 as compared to the positive control while the cells on the crosslinked MWCNT substrates released approximately 50% and 43% LDH at days 1 and 3 with no statistical differences. A statistically significant increase of 10% (p < 0.01) in LDH release was observed at day 5 timepoint LDH assay (Fig. 4B). This also corresponds to the timepoint where cell proliferation increased as observed by the MTS assay.

MTS and LDH assays were employed since they have been validated to be suitable for proliferation and cytotoxicity studies involving carbon nanoparticles^44^,^45^. MTS proliferation assay (Fig. 4A,B) indicated decreased cell proliferation at the early time point (day 1 and 3) and recovery to critical density by day 5. Previous studies show that prior to reaching a critical density, a lag phase in cell growth can occur^46^. This effect may be particularly observed when comparing flat 2D substrates, such as coverslips, and thick and rougher substrates, such as the crosslinked MWCNT coatings due to the differences in topography which can hinder proliferation of the cells. LDH cytotoxicity assay (Fig. 4C,D) indicated that cells remained comparably viable on the MWCNT substrates and the coverslip controls at days 1 and 3. The increase in LDH release at day 5 could be attributed to one of two factors: 1) an increase in basal LDH release for the increasing ADSC proliferation on crosslinked MWCNT substrates or 2) increasing cell death as the cells were in critical density without media changes for 5 days. The latter may be less likely because we did not observe an increase in LDH release for ADSCs on glass coverslips.

**Figure 5:**
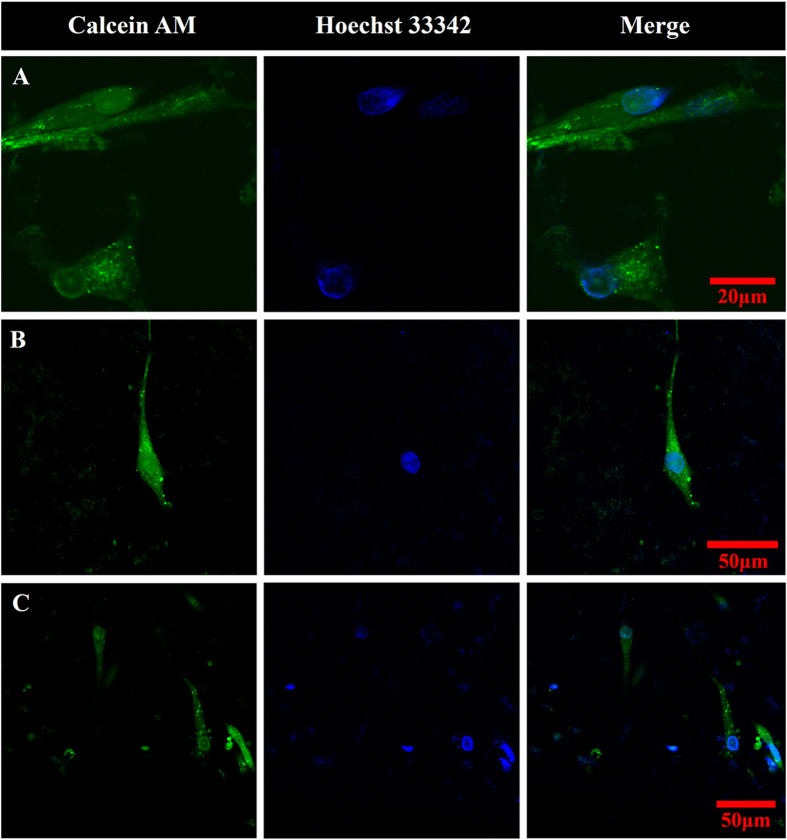
Representative confocal fluorescence microscopy of ADSCs stained with Calcein-AM (λ_ex_ = 488 nm, λ_em_ = 505 nm) and Hoechst 33342 (two-photon λ_ex_ = 800 nm, λ_em_ = 465 nm) grown on glass coverslips (**A**) and MWCNT crosslinked substrates (**B** and **C**) for 5 days at 37 °C.

Cell viability was further assessed using calcein AM live cell stain and Hoechst 33342 nuclear stain (Fig. 5). Cellular uptake of calcein AM by living cells leads to intracellular esterase cleavage, and enhanced green fluorescence due to calcein^47^. Hoechst 33342 is a nucleic acid stain which emits blue fluorescence upon binding to double-stranded DNA (dsDNA), and provides evidence on the presence of dsDNA within intact nuclear membrane of non-apoptotic cells^48^. Calcein AM verified the presence of live cells on glass coverslips (Fig. 5A) and on crosslinked MWCNT substrates (Fig. 5B). The Hoechst 33342 stains indicated that dsDNA was indeed confined within the cell in the nucleus. Together the stains provided surrogate confirmation of live cells well spread on the glass coverslips (Fig. 5A) and MWCNT substrates (Fig. 5B,C).

**Figure 6:**
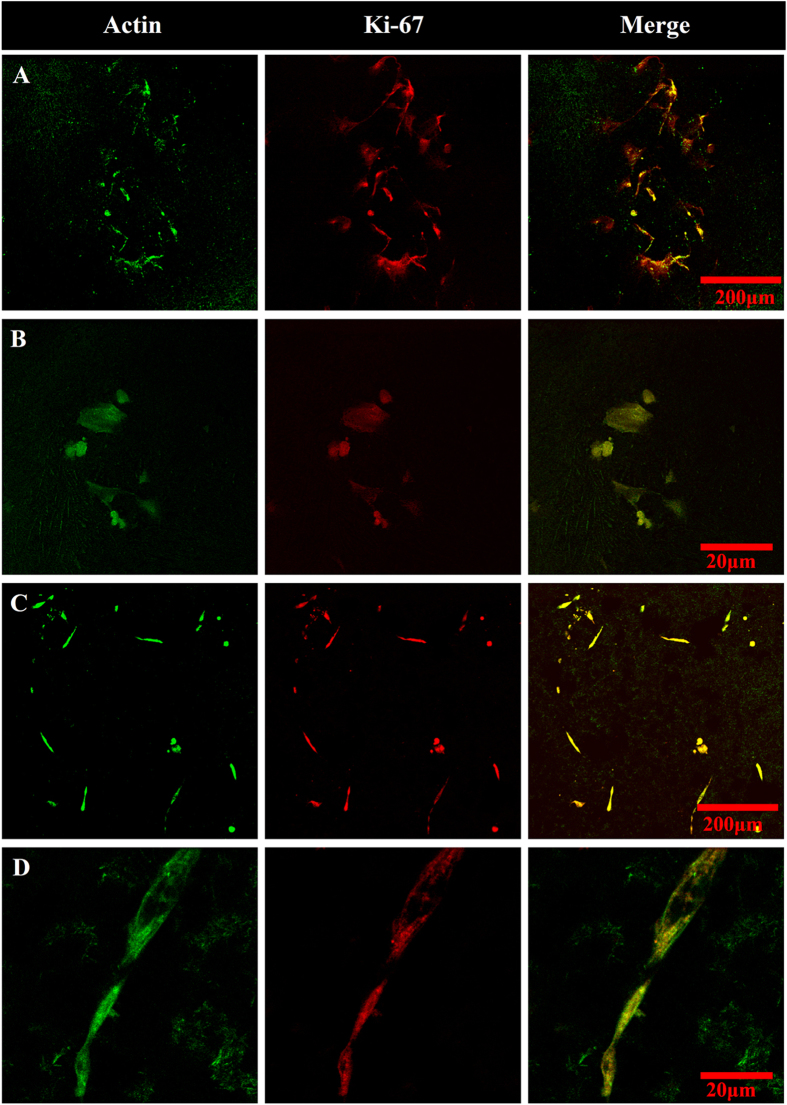
Representative confocal immunofluorescence microscopy of ADSCs for actin (λ_ex_ = 488 nm, λ_em_ = 550 nm) and proliferation marker Ki-67 (λ_ex_ = 543 nm, λ_em_ = 560 nm). Images taken for ADSCs grown for 5 days on glass coverslips at 10x (**A**) and 20x (**B**) magnification and MWCNT crosslinked substrates at 10x (**C**) and 20x magnification (**D**).

Immunochemistry studies were conducted to investigate whether cell division was arrested in cells seeded on the MWCNT substrates. In these experiments, ADSCs grown on coverslips and MWCNT films for five days were stained with fluorescently labeled antibodies for cellular proliferation marker protein, Ki-67, and probed for fluorescence (Fig. 6, center column). Ki-67 protein is present in all phases of cell growth, and can therefore be employed to evaluate whether the ADSCs are still dividing while cells that enter a G_0_ resting phase would not express Ki-67 protein^49^. Additionally, cells were stained for β-actin (Fig. 6, left column) to identify the cytoskeleton of the ADSCs. The merged images of Ki-67 and β-actin (Fig. 6, right column) show the presence of Ki-67 throughout the cell cytoplasm and nucleus for cells seeded on glass coverslips (Fig. 6A,B) or MWCNT (Fig. 6C,D) substrates implying that the cells were proliferating and metabolically active. Actin staining, also showed a more elongated cellular morphology on the MWCNT substrates (Fig. 6D) compared to the control coverslips (Fig. 6B).

**Figure 7:**
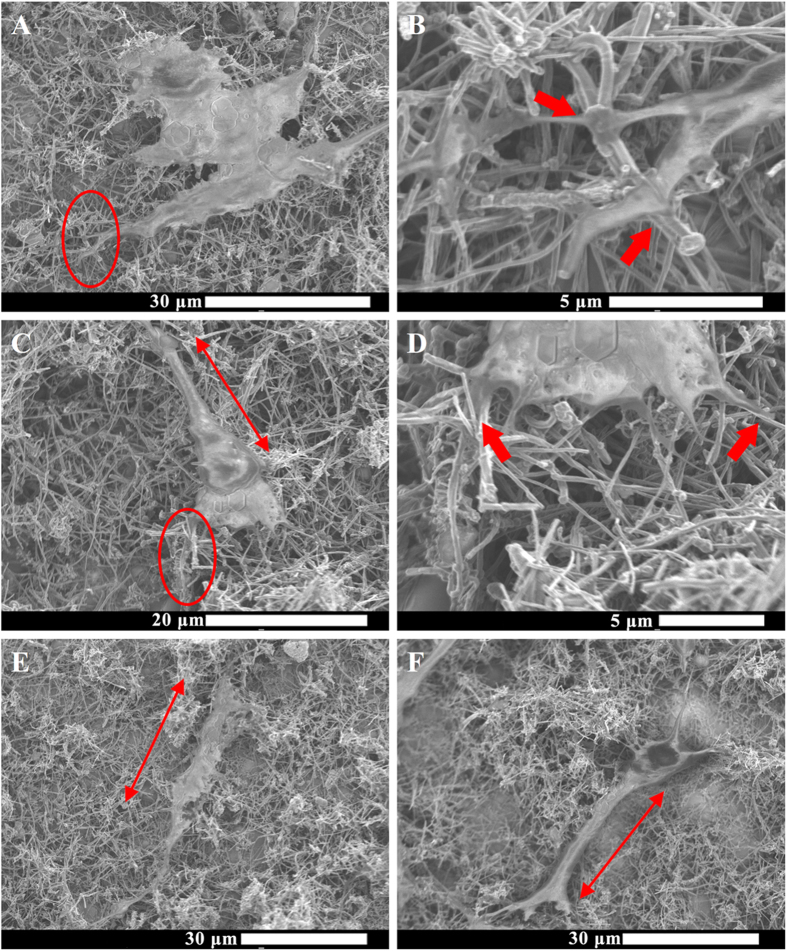
Representative SEM images of adipose derived stem cells grown on MWCNT substrates. Red circles in (**A**) and (**C**) are magnified in (**B**) and (**D**) respectively with red arrows showing cell adhesion by wrapping around nanotubes (**B**) or cell protrusions going underneath nanotube structures (**D**).

Immunohistochemistry analysis (Fig. 6) also showed cell spreading and proliferation on the MWCNT mats and provided further evidence that these mats did not affect the various phases of cell growth cycle. For further investigation, SEM was used to characterize the cellular morphology and cellular adhesion to the substrates. Figure 7 shows representative SEM images of ADSCs on the MWCNT substrates. The images shows uniaxially elongated cells (double-sided arrows in Fig. 7C,E,F) on the MWCNT substrates. The images suggested that cells interfaced well with MWCNT substrate. Cells had cytoplasmic prolongations (Fig. 7A,C red circles) that seemed to attach to the MWCNT fibers (Fig. 7B, indicated by red arrows) by wrapping over or under the fiber bundles (Fig. 7D, indicated by red arrows).

The assays, immunochemistry and SEM analysis together clearly showed that, even though the initial cell proliferation rates on the MWCNT substrates were slower compared to coverslip controls, the MWCNTs were not cytotoxic and allowed cell attachment and proliferation over the 5 day period. Harnessing the potential of carbon nanotechnology for biomedical applications often requires the 2D and 3D macroscopic assembly of nanoscale building-blocks. The results of this work introduce a novel, facile, cheap, and scalable method to fabricate robust carbon nanotube mats with chemically cross-linked junctions between sp^2^ carbon atoms, which can be easily adapted for other carbon nanostructures such as graphene and a variety of substrates with different shapes. Furthermore, with the advent of commercialized 3D printing, the mechano-structural benefits provided by the chemical crosslinks should allow for 3D-printed all-carbon nanomaterial structures.

The results open avenues further *in vitro* and *in vivo* studies to the further explore the potential of MWCNT films as a novel class of nano-fibrous mats for tissue engineering and regenerative medicine applications. The nano-fibrous MWCNT mats could be beneficial over conventional polymeric electrospun films and other methods to create nanofibrous mats^50^ for tissue engineering applications. Even though nanofibrous substrates have numerous advantages as synthetic extracellular matrix scaffolds of various tissues^51^, techniques employed to fabricate nanofibrous mats, such as molecular self-assembly, face severe commercialization and scale-up issues including expensive processing and poor control of fiber diameter^50^. Electrospinning, the current gold standard method to fabricate nanofiber requires very precise control of humidity, viscosity, and solvent volatility to produce consistent nanofibers. Marginal increases in humidity can cause large inhomogeneity in fiber diameter and clotting at the electrospray source^52^. Assembly of engineered nanomaterials such as MWCNT could overcome these fabrication challenges and allow the consistent fabrication of synthetic nanofiber mats with narrow size distribution. Further, the dense packing of electrospun fibers, and consequent lack of macro-porosity^53^, prohibiting cell infiltration into these scaffolds^54^. The method discussed herein allows fabrication of MWCNT substrates with micro- and macro porous architecture, which should allow cell infiltration. Additionally, carbon nanotubes assembled in 2D films and substrates, may exhibit multifunctional capabilities for regenerative medicine applications. The electromagnetic and electrical properties of these carbon nanostructures could be exploited to develop stimulus responsive scaffolds for electroceuticals^55^, control the fate of progenitor cells^56^,^57^,^58^, and non-invasively image the scaffolds and the regenerative process^59^.

## Conclusions

Radical initiated thermal crosslinking of carbon nanomaterials combined with air-pressure driven spray coating technique, allows rapid *in-situ* assembly of MWCNTs into chemically-crosslinked and mechanically robust MWCNT films. This protocol can be easily adapted for other carbon nanostructures such as graphene (e.g graphene nano-onions, graphene nanoribbons and graphene nanoplatelets). The crosslinked MWCNT films were found to be cytocompatible for human ADSCs. The results introduce a novel, facile, cheap, and scalable method to fabricate robust carbon nanotubes nanofiber mats and open avenues further *in vitro* and *in vivo* exploration their multifunctional potential for tissue engineering and regenerative medicine applications.

## Methods

### Film Fabrication

Multi-walled carbon nanotubes were purchased from Sigma Aldrich with the outer wall diameters of 110-170 nm and lengths of 5-9 μm. Nanoparticle suspensions at 1 mg/ml in anhydrous ethyl acetate were dispersed by sonication for 15 minutes. MWCNT to benzoyl peroxide (BP) mass ratios of 1:1, 1:2, and 1:4 were used for initial characterization. All cell studies were performed on the 1:4 ratio samples. Suspensions were sprayed with an airbrush (Iwata HP-CS) onto 12 mm diameter round glass coverslips (Electron Microscopy Sciences). Prior to spraying the coverslips were cleaned with acetone and autoclaved. During the spraying process, the coverslips were heated on a hotplate to ~60 °C to initialize *in situ* crosslinking and prevent the liquid suspension from accumulating on the surface. Samples were further thermally crosslinked in an oven at 60 °C for 12 hours. Excess BP was removed by heating the coated coverslips at 150 °C for 30 minutes.

### Surface and Chemical Characterization

Scanning electron microscopy (SEM) was performed using a JEOL 7600F analytical high resolution SEM. Samples were sputter coated with 3 nm of Au to prevent surface charge accumulation. Transmission electron microscopy (TEM) samples were prepared by fragmenting crosslinked films by scratching the surface with sharp tweezers and placing them on a conductive carbon TEM porous grid (PELCO, Ted Pella, Redding, CA). TEM was performed using a JEOL JEM2100F high resolution analytical TEM. Both electron microscopy techniques were performed at the Center for Functional Nanomaterials (Brookhaven National Laboratory, New York). For atomic force microscopy (AFM), crosslinked MWCNT (1:4) were sprayed on smooth, freshly cleaved silicon wafers (Ted Pella, Redding, CA). AFM images were obtained with a NanoSurf EasyScan 2 Flex AFM (NanoScience Instruments Inc., Phoenix AZ), in air by tapping a V-shaped cantilever (APP Nano ACL − 10, frequency fc = 145–230 kHz, L = 225 μm, W = 40 μm, tip radius <10 nm, spring constant k = 20−95 N/m). NanoSurf Easy Scan 2 Software was utilized to calculate the root mean square (r.m.s.) surface roughness of the coatings. Raman spectroscopy (Enwave Optronics, Irvine, CA) was performed in three regions of each sample (after thermal treatment to remove residual BP) under a 40x objective using a 532 nm laser source. Point spectra scanning from 100 to 3,100 cm^−1^ at room temperature were acquired.

### Mechanical Properties

The mechanical properties of spray coated pristine MWCNT and crosslinked MWCNT (MWCNT to benzoyl peroxide (BP) mass ratio of 1:4) were determined using nanoindentation (Triboindenter; Hysitron, Minneapolis, MN) with a Berkovich indenter tip. AFM specimen discs (Ted Pella) of 15 mm diameter, which can stick firmly to the magnetic triboindenter base, were coated with either MWCNT or crosslinked MWCNT and mounted into the indenter. After careful analysis of the disks under the imaging system of the triboindenter, points of indentation were selected at a distance no less than 100 μm away from each other. The imaging system of the triboindenter consisted of an objective of magnification 10X and an end zooming lens of magnification 2X. A further zoom of 5X magnification was used to decide the final selection of indentation points through the special electronically controlled magnification of the triboindenter. Samples were indented 7 times to determine elastic modulus (Er) and material hardness (H). Each indentation further comprised of 9 sub-indents in a 3 × 3 pattern and thus, the total number of indents each sample were 63. Due to the porous nature of the coatings, indents resulting in outlier points were removed individually from each 3*3 indent. Due to the porous nature of the coatings, some of the indents were not made on the thin carbon films but on the pores. Such indentations localized in holes or resulting in poor curves were not included in the analysis. The tip area function was calibrated from indentation analysis on fused quartz, and drift rates in the system were measured prior to each indentation. First, a preload of 3 μN was applied to the system followed by a constant loading rate (10 μN/second). Next, a hold segment at a fixed system load was applied, followed by a constant unloading rate to retract the tip (−10 μN/second), and finally another hold segment was applied (3 μN). Each sample was indented with peak loads ranging from ≈15 μN to 100 μN. The elastic response was calculated from the 20–90% portion of the unloading curve using methods previously described^60^. Data is reported in mean (μ), median (mdn.), standard deviation (S.D.) and interquartile range (i.q.r.).

### Electrical Characterization

Sheet resistivity was assessed by a four probe resistance measurement technique (Signatone S302-4, SP-4 probe) at Center for Functional Nanomaterials (CFN), Brookhaven National Laboratory, New York. Four spring-loaded probes, spaced equally by 1.25 mm distances, were lowered onto glass coverslips coated with crosslinked MWCNT (1:1, 1:2, and 1:4) to measure sheet resistance and resistivity.

### Cell Culture

Primary human adipose derived stem cells (ADSCs) were cultured to passage 3 in ADSC basal media supplemented with heat inactivated FBS and ADSC Growth Media Bulletkit™ (Lonza). Cells were grown in tissue culture treated polystyrene at 95% humidity, 5% CO_2_, at 37 °C with media changes every three days. Nanoparticle coated on coverslips, and plain coverslips (control) were washed with a sterile phosphate buffered saline solution (Gibco, New York) and sterilized under ultraviolet radiation for two hours. Cells were plated on the coverslips (n = 6), kept in an un-treated non-adherent 24 well plate, at a density of 4 × 10^4^ cells per well. Cells were incubated for 24 hours to allow their attachment, after which the coverslips were transferred to a new 24 well plate (considered as the Day 1 time point). Cells were kept plated for three time points; Day 1, 3 or 5. At each time point, the cells were washed twice in phosphate buffered saline solution, and used for viability and cytotoxicity assays.

### Cytotoxicity and Cytocompatibility Assays

Cytotoxicity of MWCNT 1:4 films was assessed with ADSCs by measuring lactate dehydrogenase (LDH) release from cells as a function of membrane integrity. (Sigma Aldrich; Missouri, USA). Media was collected from MWCNT films and control coverslips from each cell line at Day 1, 3 and 5. For each sample (n = 6), 200 μL of the extracted media was incubated for 45 minutes with LDH reagent and absorbance at read at 450 nm. Positive control of 100% dead cells was performed by adding 10 μl of kit-supplied lysis buffer to the control cells. Cell death was calculated from measured optical density of experimental groups, coverslip control, and positive control.

Cell proliferation of ADSCs on MWCNT 1:4 films was assessed using CellTiter 96 Cell Proliferation MTS Assay (Promega; Wisconsin, USA). Briefly, this assay is a water soluble variant of the more commonly used MTT (3-(4,5-dimethylthiazol-2-yl)-2,5-diphenyltetrazolium bromide) assay in which tetrazolium salts are converted to formazan. The colorimetric measurement of formazan (λ = 490 nm) in the experimental sample, coverslip (live) control, and positive (dead) control allows for the calculation of cell proliferation.

### Immunofluorescence/Cell Staining/SEM

Live cells were washed three times with PBS, treated with calcein-AM (0.5 mg/ml) for 30 minutes and Hoesct 33342 (2 μg/ml) for 30 minutes. For immunofluorescence microscopy, glutaraldehyde fixed cells were washed with PBS, incubated with 2% glycine for 5 minutes, and permeabilized using 0.5% Triton-X-100 permeabilizing buffer (10.3 g sucrose, 0.4 g Hepes buffer, 0.29 g NaCl, 0.06 g MgCl_2_, and 0.5 ml Triton-X-100 in 100 ml DI water) for 25 minutes. Samples were washed using immunofluorescence buffer (IFB, 0.1% BSA and 0.1% Triton-X-100 in PBS) and incubated with commercially available monoclonal anti-proliferating Ki-67 antibody raised in mouse (2 μl/ml in IFB, Cat. No. P8825, Sigma Aldrich, New York, USA) for 1 hour. Samples were washed with IFB (3X) and incubated with anti-mouse rhodamine conjugated secondary antibody (2 μl/ml in IFB, Cat. No. SAB3701218, Sigma Aldrich, New York, USA) for 1 hour. Samples were washed with IFB (3X) and stained with FITC conjugated phalloidin (2 μl/ml in PBS) for 1 hour to visualize cytoskeleton (actin filaments). Samples were then imaged using a confocal laser scanning microscope (Zeiss LSM 510 Two-Photon LSCM).

Specimens for scanning electron microscopy (SEM) were prepared as follows. MWCNT 1:4 samples with ADSCs were dehydrated by serial ethanol wetting steps from 50% to anhydrous ethanol. The samples were then air dried for one day and vacuum dried overnight at room temperature. A 3 nm layer of gold sputter was applied prior to SEM. SEM was performed on a high resolution analytical JOEL 7600F SEM at the Center for Functional Nanomaterials (Brookhaven National Laboratories).

### Statistical Analysis

All plots for cell studies present a mean and standard deviation. Statistical analysis for cell studies was performed with one-way ANOVA followed by Tukey-Kramer post hoc analysis (Graphpad Prism). Statistical analysis for nanoindentation was performed using Mann-Whitney test (Graphpad Prism). A 95% confidence interval (*p* < 0.05) was used for all statistical analysis.

## Additional Information

**How to cite this article**: Patel, S. C. *et al.* Fabrication and Cytocompatibility of *In Situ* Crosslinked Carbon Nanomaterial Films. *Sci. Rep.* **5**, 10261; doi: 10.1038/srep10261 (2015).

## Supplementary Material

Supplementary Information

## Figures and Tables

**Table 1:** Mechanical properties of spray coated pristine MWCNT and crosslinked MWCNT (MWCNT: BP = 1:4) determined by nanoindentation.

	**PRISTINE MWCNT**		**CROSSLINKED MWCNT**	
	**Er (MPa)**	**H (MPa)s**	**Er (MPa)**	**H (MPa)**
Median	162^#^	2.13*	376^#^	5.15*
I.Q.R.	173	1.53	307	5.16
min.	20.7	0.452	59.8	0.881
max	1762	8.87	1604	22.3

(*represents p < 0.0001 between groups and ^#^represents p < 0.0001 between groups)

## References

[b1] Lalwani G. & Sitharaman B. Multifunctional fullerene-and metallofullerene-based nanobiomaterials. Nano LIFE 3, 1342003-1-22 (2013).

[b2] Zhang Y., Nayak T. R., Hong H. & Cai W. Graphene: a versatile nanoplatform for biomedical applications. Nanoscale 4, 3833–3842 (2012). 22653227 10.1039/c2nr31040f PMC3376191

[b3] Harrison B. & Atala A. Carbon nanotube applications for tissue engineering. Biomaterials 28, 344–353 (2007). 16934866 10.1016/j.biomaterials.2006.07.044

[b4] Jariwala D., Sangwan V. K., Lauhon L. J., Marks T. J. & Hersam M. C. Carbon nanomaterials for electronics, optoelectronics, photovoltaics, and sensing. Chem. Soc. Rev. 42, 2824–2860 (2013). 23124307 10.1039/c2cs35335k

[b5] Nayak T. R. *et al.* Graphene for controlled and accelerated osteogenic differentiation of human mesenchymal stem cells. ACS Nano 5, 4670–4678 (2011). 21528849 10.1021/nn200500h

[b6] Ryoo S.-R., Kim Y.-K., Kim M.-H. & Min D.-H. Behaviors of NIH-3T3 fibroblasts on graphene/carbon nanotubes: proliferation, focal adhesion, and gene transfection studies. ACS Nano 4, 6587–6598 (2010). 20979372 10.1021/nn1018279

[b7] Hirata E. *et al.* Multiwalled carbon nanotube-coating of 3D collagen scaffolds for bone tissue engineering. Carbon 49, 3284–3291 (2011).

[b8] Hu L., Hecht D. S. & Gruner G. Carbon nanotube thin films: fabrication, properties, and applications. Chem. Rev. 110, 5790–5844 (2010). 20939616 10.1021/cr9002962

[b9] Lobo A. *et al.* Cell viability and adhesion on as grown multi-wall carbon nanotube films. Mater. Sci. Eng. C 28, 264–269 (2008).

[b10] Kalbacova M., Broz A., Kong J. & Kalbac M. Graphene substrates promote adherence of human osteoblasts and mesenchymal stromal cells. Carbon 48, 4323–4329 (2010).

[b11] Chen H., Müller M. B., Gilmore K. J., Wallace G. G. & Li D. Mechanically strong, electrically conductive, and biocompatible graphene paper. Adv. Mater. 20, 3557–3561 (2008).

[b12] Lau K. K. *et al.* Superhydrophobic carbon nanotube forests. Nano Lett. 3, 1701–1705 (2003).

[b13] Reina A. *et al.* Large area, few-layer graphene films on arbitrary substrates by chemical vapor deposition. Nano Lett. 9, 30–35 (2008). 19046078 10.1021/nl801827v

[b14] Wu Z. *et al.* Transparent, conductive carbon nanotube films. Science 305, 1273–1276 (2004). 15333836 10.1126/science.1101243

[b15] Nayak T. R. *et al.* Thin films of functionalized multiwalled carbon nanotubes as suitable scaffold materials for stem cells proliferation and bone formation. ACS Nano 4, 7717–7725 (2010). 21117641 10.1021/nn102738c

[b16] Tutak W. *et al.* Toxicity induced enhanced extracellular matrix production in osteoblastic cells cultured on single-walled carbon nanotube networks. Nanotechnology 20, 255101 (2009). 19487801 10.1088/0957-4484/20/25/255101

[b17] Lalwani G. *et al.* Fabrication and characterization of three-dimensional macroscopic all-carbon scaffolds. Carbon 53, 90–100 (2012). 23436939 10.1016/j.carbon.2012.10.035 PMC3578711

[b18] Dresselhaus M. S., Dresselhaus G., Saito R. & Jorio A. Raman spectroscopy of carbon nanotubes. Phys. Rep. 409, 47–99 (2005).

[b19] Sahoo S., Chakraborti C., Mishra S., Naik S. & Nanda U. FTIR and Raman Spectroscopy as a Tool for Analyzing Sustained Release Hydrogel of Ciprofloxacin/Carbopol Polymer. J. Adv. Pharm. Technol. Res. 2, 195–204 (2011).22171318

[b20] Nirmalraj P. N., Lyons P. E., De S., Coleman J. N. & Boland J. J. Electrical Connectivity in Single-Walled Carbon Nanotube Networks. Nano Lett. 9, 3890–3895 (2009). 19775126 10.1021/nl9020914

[b21] Murakami Y. *et al.* Growth of vertically aligned single-walled carbon nanotube films on quartz substrates and their optical anisotropy. Chem. Phys. Lett. 385, 298–303 (2004).

[b22] Dikin D. A. *et al.* Preparation and characterization of graphene oxide paper. Nature 448, 457–460 (2007). 17653188 10.1038/nature06016

[b23] Xu G., Zhang Q., Zhou W., Huang J. & Wei F. The feasibility of producing MWCNT paper and strong MWCNT film from VACNT array. App. Phys. A 92, 531–539 (2008).

[b24] Kaempgen M., Duesberg G. & Roth S. Transparent carbon nanotube coatings. App. Surf. Sci. 252, 425–429 (2005).

[b25] Keefer E. W., Botterman B. R., Romero M. I., Rossi A. F. & Gross G. W. Carbon nanotube coating improves neuronal recordings. Nat. Nanotechnol. 3, 434–439 (2008). 18654569 10.1038/nnano.2008.174

[b26] Collaert N. *et al.* *In vitro* recording of neural activity using carbon nanosheet microelectrodes. Carbon 67, 178–184 (2014).

[b27] Wang Z., Koratkar N., Ci L. & Ajayan P. M. Combined micro-/nanoscale surface roughness for enhanced hydrophobic stability in carbon nanotube arrays. App. Phys. Lett. 90, 143117 (2007).

[b28] Tenent R. C. *et al.* Ultrasmooth, Large‐Area, High‐Uniformity, Conductive Transparent Single‐Walled‐Carbon‐Nanotube Films for Photovoltaics Produced by Ultrasonic Spraying. Adv. Mater. 21, 3210–3216 (2009).

[b29] Yang J., Zhang Z., Men X. & Xu X. Fabrication of stable, transparent and superhydrophobic nanocomposite films with polystyrene functionalized carbon nanotubes. App. Surf. Sci. 255, 9244–9247 (2009).

[b30] Geng H.-Z. *et al.* Effect of acid treatment on carbon nanotube-based flexible transparent conducting films. J. Am. Chem. Soc. 129, 7758–7759 (2007). 17536805 10.1021/ja0722224

[b31] Xue W. & Cui T. Characterization of layer-by-layer self-assembled carbon nanotube multilayer thin films. Nanotechnology 18, 145709 (2007).

[b32] Cha S. I. *et al.* Mechanical and electrical properties of cross-linked carbon nanotubes. Carbon 46, 482–488 (2008).

[b33] Zhang L., Zhang G., Liu C. & Fan S. High-Density Carbon Nanotube Buckypapers with Superior Transport and Mechanical Properties. Nano Lett. 12, 4848–4852, (2012). 22925031 10.1021/nl3023274

[b34] Dai H., Wong E. W. & Lieber C. M. Probing electrical transport in nanomaterials: conductivity of individual carbon nanotubes. Science 272, 523–526 (1996).

[b35] Zhang M. *et al.* Strong, transparent, multifunctional, carbon nanotube sheets. Science 309, 1215–1219 (2005). 16109875 10.1126/science.1115311

[b36] Kaempgen M., Duesberg G. S. & Roth S. Transparent carbon nanotube coatings. App. Surf. Sci. 252, 425–429 (2005).

[b37] Bateman L. Olefin oxidation. Q. Rev. Chem. Soc. 8, 147–167 (1954).

[b38] Stankovich S. *et al.* Synthesis of graphene-based nanosheets via chemical reduction of exfoliated graphite oxide. Carbon 45, 1558–1565 (2007).

[b39] Fernandez-Merino M. *et al.* Vitamin C is an ideal substitute for hydrazine in the reduction of graphene oxide suspensions. J. Phys. Chem. C 114, 6426–6432 (2010).

[b40] Talukdar Y., Rashkow J. T., Lalwani G., Kanakia S. & Sitharaman B. The effects of graphene nanostructures on mesenchymal stem cells. Biomaterials 35, 4863–4877 (2014). 24674462 10.1016/j.biomaterials.2014.02.054 PMC3995421

[b41] Kern S., Eichler H., Stoeve J., Klüter H. & Bieback K. Comparative analysis of mesenchymal stem cells from bone marrow, umbilical cord blood, or adipose tissue. Stem Cells 24, 1294–1301 (2006). 16410387 10.1634/stemcells.2005-0342

[b42] Yanez R. *et al.* Adipose Tissue‐Derived Mesenchymal Stem Cells Have *In Vivo* Immunosuppressive Properties Applicable for the Control of the Graft‐Versus‐Host Disease. Stem Cells 24, 2582–2591 (2006). 16873762 10.1634/stemcells.2006-0228

[b43] Lewinski N., Colvin V. & Drezek R. Cytotoxicity of nanoparticles. Small 4, 26–49 (2008). 18165959 10.1002/smll.200700595

[b44] Mullick Chowdhury S. *et al.* Cell specific cytotoxicity and uptake of graphene nanoribbons. Biomaterials 34, 283–293 (2012). 23072942 10.1016/j.biomaterials.2012.09.057 PMC3489471

[b45] Avti P. K., Caparelli E. D. & Sitharaman B. Cytotoxicity, cytocompatibility, cell‐labeling efficiency, and *in vitro* cellular magnetic resonance imaging of gadolinium‐catalyzed single‐walled carbon nanotubes. J. Biomed. Mater. Res. A 101, 3580–3591 (2013). 23686792 10.1002/jbm.a.34643 PMC3785562

[b46] Bensaïd W. *et al.* A biodegradable fibrin scaffold for mesenchymal stem cell transplantation. Biomaterials 24, 2497–2502 (2003). 12695076 10.1016/s0142-9612(02)00618-x

[b47] Sayes C. M. *et al.* The differential cytotoxicity of water-soluble fullerenes. Nano Lett. 4, 1881–1887 (2004).

[b48] Allen S., Sotos J., Sylte M. & Czuprynski C. Use of Hoechst 33342 staining to detect apoptotic changes in bovine mononuclear phagocytes infected with Mycobacterium avium subsp. paratuberculosis. Clin. Diagn. Lab. Immunol. 8, 460–464 (2001). 11238240 10.1128/CDLI.8.2.460-464.2001 PMC96081

[b49] Scholzen T. & Gerdes J. The Ki‐67 protein: from the known and the unknown. J. Cell. Physiol. 182, 311–322 (2000). 10653597 10.1002/(SICI)1097-4652(200003)182:3<311::AID-JCP1>3.0.CO;2-9

[b50] Luo C., Stoyanov S. D., Stride E., Pelan E. & Edirisinghe M. Electrospinning versus fibre production methods: from specifics to technological convergence. Chem. Soc. Rev. 41, 4708–4735 (2012). 22618026 10.1039/c2cs35083a

[b51] Stevens M. M. & George J. H. Exploring and engineering the cell surface interface. Science 310, 1135–1138 (2005). 10.1126/science.1106587 16293749

[b52] Subbiah T., Bhat G., Tock R., Parameswaran S. & Ramkumar S. Electrospinning of nanofibers. J. Appl. Polym. Sci. 96, 557–569 (2005).

[b53] Baker B. M. *et al.* The potential to improve cell infiltration in composite fiber-aligned electrospun scaffolds by the selective removal of sacrificial fibers. Biomaterials 29, 2348–2358 (2008). 18313138 10.1016/j.biomaterials.2008.01.032 PMC2292637

[b54] Hollister S. J. Porous scaffold design for tissue engineering. Nat. Mater. 4, 518–524 (2005). 16003400 10.1038/nmat1421

[b55] Famm K., Litt B., Tracey K. J., Boyden E. S. & Slaoui M. Drug discovery: A jump-start for electroceuticals. Nature 496, 159–161 (2013). 23579662 10.1038/496159a PMC4179459

[b56] Supronowicz P. *et al.* Novel current‐conducting composite substrates for exposing osteoblasts to alternating current stimulation. J. Biomed. Mater. Res. A 59, 499–506 (2002). 10.1002/jbm.10015 11774308

[b57] Harrison B. S. & Atala A. Carbon nanotube applications for tissue engineering. Biomaterials 28, 344–353 (2007). 16934866 10.1016/j.biomaterials.2006.07.044

[b58] Sitharaman B., Avti P. K., Schaefer K., Talukdar Y. & Longtin J. P. A novel nanoparticle-enhanced photoacoustic stimulus for bone tissue engineering. Tissue Eng. Part A 17, 1851–1858 (2011). 21395444 10.1089/ten.tea.2010.0710 PMC3118605

[b59] Pramanik M., Swierczewska M., Wang L. V., Green D. & Sitharaman B. Single-walled carbon nanotubes as a multimodal-thermoacoustic and photoacoustic-contrast agent. J. Biomed. Opt. 14, 034018-034018-034018 (2009). 10.1117/1.3147407 PMC2732201 19566311

[b60] Ozcivici E., Ferreri S., Qin Y.-X. & Judex S. Determination of bone’s mechanical matrix properties by nanoindentation. Osteoporosis 323–334 (Springer, 2008). 10.1007/978-1-59745-104-8_22 18463828

