# Supplementary material for: Fabrication and Cytocompatibility of In Situ Crosslinked Carbon Nanomaterial Films
Source: Sci Rep. 2015 May 28;5:10261. doi: 10.1038/srep10261 (PMC4446896; doi:10.1038/srep10261)
Supplement: Supplementary Information [file srep10261-s1.pdf]

Fabrication and Cytocompatibility of *In Situ* Crosslinked Carbon Nanomaterial Films

Supplemental Information

Sunny C. Patel, Gaurav Lalwani, Kartikey Grover, Yi-Xian Qin, Balaji Sitharaman*

Department of Biomedical Engineering, Stony Brook University, Stony Brook, New York 11794-5281

*Correspondence:

Balaji Sitharaman, Ph.D.

Department of Biomedical Engineering

Bioengineering Building Room 115

Stony Brook University

Stony Brook, NY 11794-5281

Tel: 631-632-1810

Email: balaji.sitharaman@stonybrook.edu

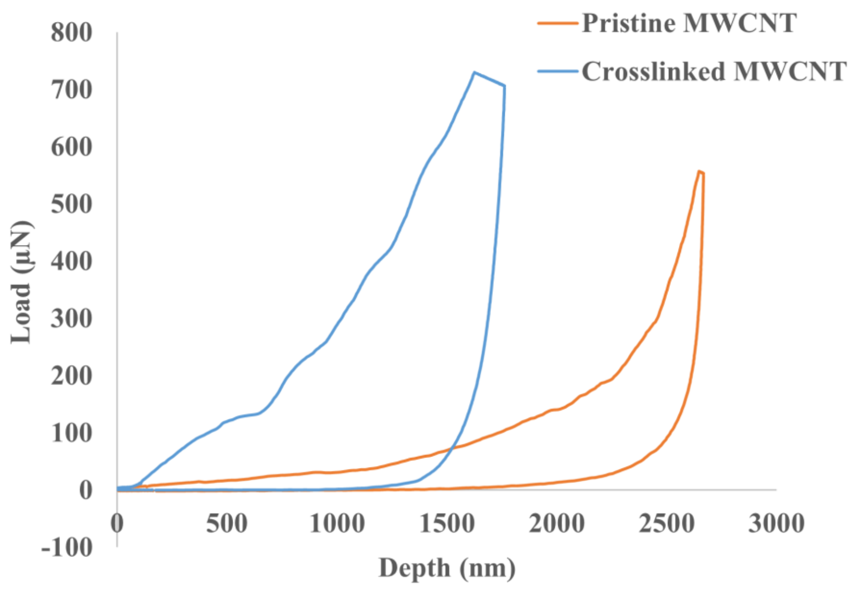

Supplementary Figure 1. Representative load-unloading curve from nanoindentation of spray coated non-crosslinked pristine MWCNT and crosslinked MWCNT (1:4). Elastic modulus (Er) was calculated from the unloading region of each curve.

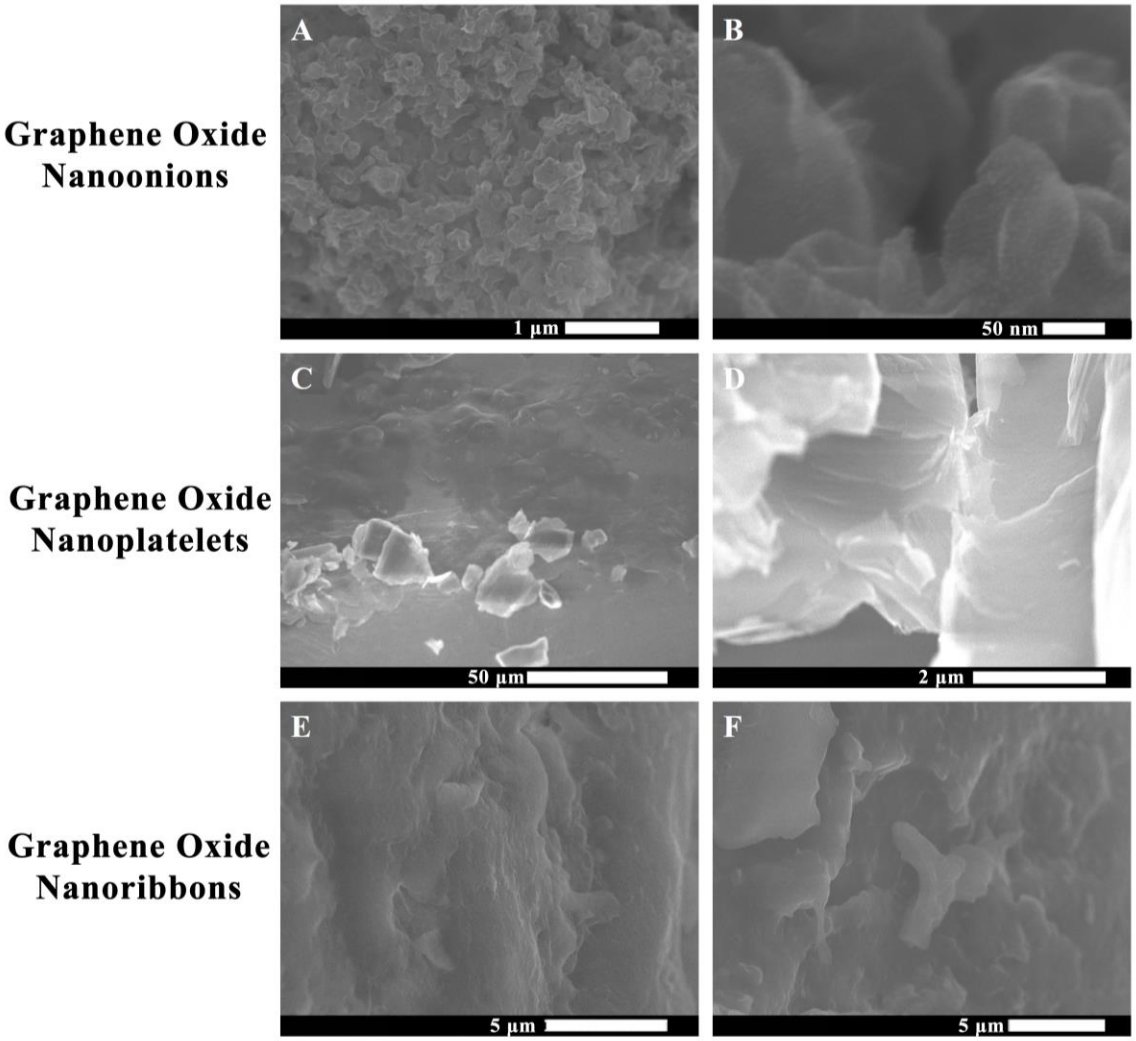

Supplementary Figure 2. Representative low-magnification crosslinked (A, C, E) GONO, GONP, and GONR, and high-magnification crosslinked (B, D, F) GONO, GONP, and GONR SEM images.
